# Heat Capacities and Thermodynamic Properties of Hungchaoite and Mcallisterite

**DOI:** 10.3390/molecules24244470

**Published:** 2019-12-06

**Authors:** Jiangtao Song, Fei Yuan, Long Li, Yafei Guo, Tianlong Deng

**Affiliations:** Tianjin Key Laboratory of Marine Resources and Chemistry, College of Chemical Engineering and Materials Science, Tianjin University of Science and Technology, Tianjin 300457, Chinalilong@tust.edu.cn (L.L.); tldeng@tust.edu.cn (T.D.)

**Keywords:** thermodynamic function, heat capacity, hungchaoite, mcallisterite, calorimetry

## Abstract

The heat capacities on two minerals of hungchaoite (MgB_4_O_7_·9H_2_O, Hu) and mcallisterite (MgB_6_O_10_·7.5H_2_O, Mc) have been measured with a precision calorimeter at temperatures ranging from 306.15 to 355.15 K, experimentally. It was found that there are no phase transition and thermal anomalies, and the molar heat capacities against temperature for the minerals of hungchaoite and mcallisterite were fitted as $C_{p,m,\mathrm{Hu}\;}=\;-27019.23675+229.55286T\;-\;{0.63912T}^{\;2}\;+\;({5.95862\;\times \;10}^{\;-4}{)\;T}^{\;3}$ and $C_{p,\mathrm{mMc}\;}=\;-9981.88552\;+\;84.10964T\;-\;{0.22685T}^{\;2}\;+\;({2.0593\;\times \;10}^{\;-4}{)\;T}^{\;3}$, respectively. The molar heat capacities and thermodynamic functions of (*H_T_*-*H*_298.15_), (*S_T_*-*S*_298.15_), and (*G_T_*-*G*_298.15_) at intervals of 1 K for the two minerals were obtained for the first time. These results are significant in order to understand the thermodynamic properties of those minerals existing in nature salt lakes, as well as applying them to the chemical engineering process design.

## 1. Introduction

Magnesium borates and their superior performances are widely used in chemical industries, alkali-free glass fiber, ceramic, and agriculture [1,2]. China is rich in boron resources with solid boron mineral in the east and liquid boron mineral in the brine of salt lakes in the west. Especially, the Qinghai-Tibet Plateau Salt Lake is famous for abundant boron-magnesium resources [3,4], and it has been found to have 14 kinds of natural borate minerals [5,6]. Among them, several kinds of the most common magnesium borate hydrates, which are pinnoite (MgB_2_O_4_·3H_2_O), mcallisterite (MgB_6_O_10_·7.5H_2_O), hungchaoite (MgB_4_O_7_·9H_2_O), inderite (Mg_2_B_6_O_11_·15H_2_O), and kurnakovite (Mg_2_B_6_O_11_·15H_2_O) [7,8,9,10,11,12]. However, the thermodynamic properties on these normal magnesium borates are scarce. Therefore, studies on the thermodynamic properties of different forms of magnesium borates are essential to provide important basic thermodynamic data for the development and utilization of the salt lake brine resources in the western region of China.

Heat capacity means the basic thermodynamic property, which can reflect the interaction between solute and solvent in the solution system [13,14]. The apparent molar enthalpy at different concentrations was calculated by the heat capacity at different temperatures. In addition, it is also one of the essential basic data of chemical production [15,16]. According to the heat capacity data, accurate information about the materialized treatment and reaction device design can be obtained [17,18]. At the same time, basic properties such as enthalpy, entropy, and Gibbs free energy can be calculated by heat capacity, and the structure and phase transition can also be analyzed by it.

The heat capacity and thermodynamic properties of magnesium borates are basic thermodynamic parameters, which provide basic data for the comprehensive utilization of magnesium and boron resources. According to the Tarasov theory of the heat capacity of solids [13], which is a particular case of the fractal heat capacity theory, the dependence of T is proportional to T for solids with chain structures, T2 for layer solids, and T3 for framework structures.

In this research, the heat capacities and thermodynamic properties on two minerals of hungchaoite (MgB_4_O_7_·9H_2_O) and mcallisterite (MgB_6_O_10_·7.5H_2_O) in the temperature range of 306.15~365.15 K were measured by a precision calorimeter for the first time.

## 2. Experimental

### 2.1. Chemicals

(MgCO_3_)_4_·Mg(OH)_2_·5H_2_O (A.R., Macklin Chemical Reagent Co., Ltd., Shanghai, China); H_3_BO_3_ (A.R., Sinopharm Chemical Reagent Co., Ltd., Shanghai, China); double deionized water (DDW) with its conductivity less than 0.055 μS·m−1 was produced by the deionized water machine (ULUP-II-10T, Chongqing Jiuyang Co. Lt., Chongqing, China).

### 2.2. Synthesis 

Hungchaoite (MgB_4_O_7_·9H_2_O) and mcallisterite (MgB_6_O_10_·7.5H_2_O) were synthesized according to the reference [19]. Briefly, hungchaoite and mcallisterite were both synthesized with basic magnesium carbonate, magnesium oxide, and boric acid. Initially, active magnesium oxide was made from basic magnesium carbonate in a Muffle Furnace (SX2-5-12, Tianjing Zhonghuan Co., Tianjin, China) at 873.15 K for 6 h. Then, magnesium oxide, basic magnesium carbonate, and boric acid were dissolved at 298.15 K and stirred for 3.5 h to filter the liquid. The magnetic stirring was paused for 4 h. Finally, the solid phase was separated by washing three times with absolute ethyl alcohol and DDW separately. All the samples were dried in a desiccator before using.

### 2.3. Identification and Analytical Methods

**Figure 1:**
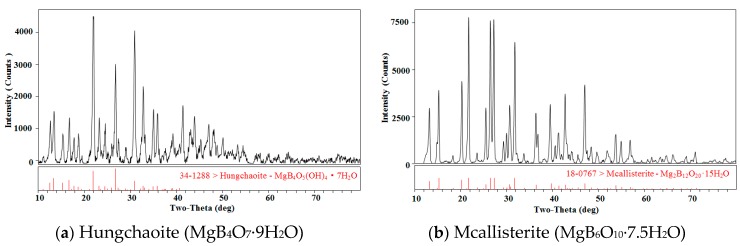
The X-ray diffraction patterns for minerals hungchaoite and mcallisterite. The codes “34-1288” and “18-0767” denote standard codes of X-ray of hungchaoite and mcallisterite.

MgB_4_O_7_·9H_2_O and MgB_6_O_10_·7.5H_2_O were identified by X-ray diffractometer (MSAL XD-3, Beijing Purkinje General Instrument Co., Ltd., Beijing, China) with Cu-Kα radiation at 4°·min^−1^, and the results are shown in Figure 1.

**Figure 2:**
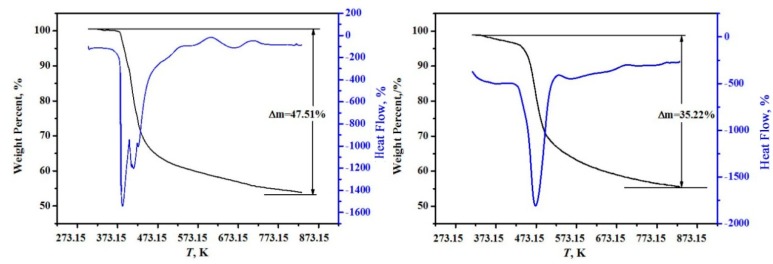
The high-precision thermal analysis (TG-DSC) curves of hungchaoite (**on the left**) and mcallisterite (**on the right**).

The high-precision thermal analysis (TG-DSC, Labsys, Setaram, France) under argon atmosphere with a heating rate of 10 K/min was used, and the TG-DSC curves for the two minerals are shown in Figure 2. It can be found from Figure 2 that the samples were continuously weightless at 368.15~748.15 K, the total weightlessness rates were 47.51% and 35.22% for MgB_4_O_7_·9H_2_O and MgB_6_O_10_·7.5H_2_O, respectively. A comparison of dehydrated crystal waters between the theoretical values and the experimental values agree well and the deviations are within 0.17% in Table 1.

The content of magnesium was determined by EDTA titration with an uncertainty of 0.003 [20], and the content of B2O3 was analyzed by the method of mannitol weight titration with an uncertainty of 0.0005 [21]. The chemical analysis results for MgB_4_O_7_·9H_2_O and MgB_6_O_10_·7.5H_2_O are presented in Table 1.

### 2.4. Calorimetry and Experiment Method

The high-precision calorimeter (TG-DSC Labsys, Setaram, France) was used for a heat capacity experiment, which requires three groups of experiments, namely, blank experiment, reference experiment, and sample experiment, and Alumina as reference experiment. To verify the performance, the heat capacity of KCl was measured, and the average experimental value for five times of 0.6860 J·g^−1^·K^−1^ is in accordance with 0.6879 J·g^−1^·K^−1^ reported in the literature [22], whose deviation is 0.28%. The heat capacities were carried out from 303 K to 355 K with a heating rate of 5 K/min and a flow rate of N_2_ is 20 mL/min, and weighting 16.02 ± 0.01 and 16.65 ± 0.01 mg samples of hungchaoite and mcallisterite were used, respectively.

## 3. Results and Discussion

### 3.1. Heat Capacities

**Figure 3:**
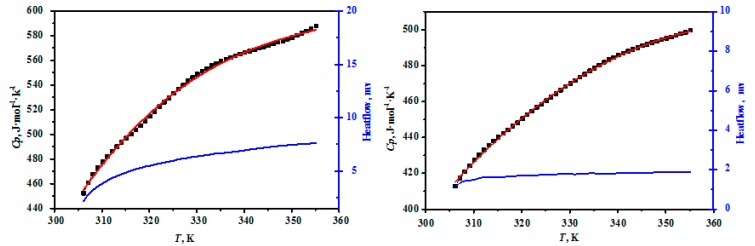
Comparisons on the experimental and fitting results for the compounds of hungchaoite (**on the left**) and mcallisterite (**on the right**) at temperatures ranging from 306.15 to 355.15 K.

The heat capacities of MgB_4_O_7_·9H_2_O and MgB_6_O_10_·7.5H_2_O are presented in Table 2 and the comparisons between the experimental and fitting results for the compounds were plotted in Figure 3. As can be seen in Figure 3, the heat capacity curves of the hungchaoite and mcallisterite increase slowly with the increasing temperature, which showed that the structure of the hungchaoite and mcallisterite were smooth within the temperature range. There is no phase change, association, and thermal decomposition occurring from 306.15 K to 355.15 K for hungchaoite and mcallisterite. Molar heat capacities polynomial equations of hungchaoite and mcallisterite are expressed as Equations (1) and (2), respectively.

(1) $$C_{p,\;m,\;\mathrm{Hu}}\;=\;-27019.23675\;+\;229.55286T\;-\;{0.63912T}^{\;2}\;+\;({5.95862\;\times \;10}^{-4}{)\;T}^{3}$$

(2) $$C_{p,\;m\;\mathrm{Mc}\;}=\;-9981.88552\;+\;84.10964T\;-\;{0.22685T}^{2}\;+\;({2.0593\text{×}10}^{-4}{)\;T}^{3}$$

**Figure 4:**
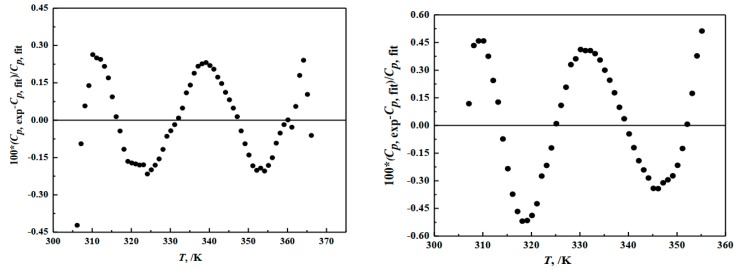
Deviation of heat capacities of the compounds of hungchaoite (**on the left**) and mcallisterite (**on the right**) between experimental *C*_p, exp_ and calculated *C*_p, fit._

The regression analysis results are shown in Table 2 and Figure 4 and the determination coefficient of the fitting curves are $R_{\mathrm{Hu}}^{2}$ = 0.9975 and $R_{\mathrm{Mc}}^{2}$ = 0.9992. The deviation between the fitting values and the experimental values of hungchaoite and mcallisterite are 0.32% and 1.8%, separately. The relative deviation between the fitting value and the experimental value of two compounds are both less than 0.47%.

### 3.2. Enthalpy, Entropy, and Gibbs Free Energy

The molar heat capacity is calculated according to the fitting heat capacity curve. The standard reference temperature of the thermodynamic function is 298.15 K, (*H*_T_-*H*_298.15_), (*S*_T_-*S*_298.15_), and (*G*_T_-*G*_298.15_) calculated according to the following thermodynamic functions. The values of (*H*_T_-*H*_298.15_), (*S*_T_-*S*_298.15_), and (*G*_T_-*G*_298.15_) for the hungchaoite and mcallisterite are listed in Table 3.

(3) $$H(T)\;-\;H(298.15\;K)\;=\;\int_{298.15}^{T}Cp,m\;dT,$$

(4) $$S(T)\;-\;S(298.15\;K)\;=\;\int_{298.15}^{T}\frac{Cp,m}{T}dT,$$

(5) $$G(T)\;-\;G(298.15\;K)\;=\;\int_{298.15}^{T}Cp,m\;dT\;-\;T\;\int_{298.15}^{T}\frac{Cp,m}{T}dT.$$

## 4. Conclusions

The heat capacities of hungchaoite (MgB_4_O_7_·9H_2_O) and mcallisterite (MgB_6_O_10_·7.5H_2_O) were measured using the precision calorimetry, and the molar heat capacities of hungchaoite and mcallisterite were derived experimentally. The experimental results showed that the molar heat capacities of the two minerals are increased with the increasing of temperature, and no phase change, association, and thermal decomposition were found at temperatures ranging from 306.15 to 355.15 K. At the same time, the polynomial equations of the molar heat capacities against the temperature for the two minerals were fitted by the least square method. The thermodynamic functions of (*H_T_*-*H*_298.15_), (*S_T_*-*S*_298.15_), and (*G_T_*-*G*_298.15_) at intervals of 1 K for the two minerals were obtained for the first time.

## Figures and Tables

**Table 1:** Analytical results of hungchaoite and mcallisterite.

Component Content	MgO/wt %	B_2_O_3_/wt %	H_2_O/wt %	*n* (MgO:B_2_O_3_:H_2_O)
Hungchaoite, MgB_4_O_7_·9H_2_O				
Experimental value	11.86	40.79	47.35	1:2.04:8.95
Theoretical value	11.80	40.75	47.45	1:2.00:9.00
Relative error (%)	0.51	0.05	0.20	-
Mcallisterite, MgB_6_O_10_·7.5H_2_O				
Experimental value	10.50	54.60	34.87	1:3.00:7.42
Theoretical value	10.49	54.35	35.16	1:3.00:7.50
Relative error (%)	0.10	0.46	0.82	-

**Table 2:** Experimental values of molar heat capacities of hungchaoite (MgB_4_O_7_·9H_2_O) and mcallisterite (MgB_6_O_10_·7.5H_2_O) at 306.15~355.15 K and 101.325 kPa ^a^.

$\frac{T}{K}\;$	$\frac{Cp}{J\text{·}{\mathrm{mol}}^{-1}\text{·}K^{-1}}$	$\frac{T}{K}$	$\frac{Cp}{J\text{·}{\mathrm{mol}}^{-1}\text{·}K^{-1}}$	$\frac{T}{K}$	$\frac{Cp}{J\text{·}{\mathrm{mol}}^{-1}\text{·}K^{-1}}$
Hungchaoite, MgB_4_O_7_·9H_2_O					
306.15	452.21	323.15	525.99	340.15	566.32
307.15	460.92	324.15	529.57	341.15	567.48
308.15	467.61	325.15	533.26	342.15	568.58
309.15	472.84	326.15	536.68	343.15	569.74
310.15	477.79	327.15	539.99	344.15	570.90
311.15	482.20	328.15	543.34	345.15	571.92
312.15	486.20	329.15	546.07	346.15	573.19
313.15	490.12	330.15	548.84	347.15	574.59
314.15	493.50	331.15	551.19	348.15	575.85
315.15	496.89	332.15	553.48	349.15	577.12
316.15	500.27	333.15	555.60	350.15	578.55
317.15	503.72	334.15	557.54	351.15	580.12
318.15	507.23	335.15	559.25	352.15	581.90
319.15	510.92	336.15	560.93	353.15	583.84
320.15	514.61	337.15	562.39	354.15	586.00
321.15	518.37	338.15	563.76	355.15	587.70
322.15	522.47	339.15	565.13		
Mcallisterite, MgB_6_O_10_·7.5H_2_O					
306.15	412.82	323.15	456.70	340.15	485.82
307.15	417.28	324.15	458.50	341.15	486.97
308.15	420.89	325.15	460.50	342.15	488.04
309.15	424.16	326.15	462.46	343.15	489.08
310.15	427.54	327.15	464.38	344.15	490.08
311.15	430.26	328.15	466.34	345.15	491.04
312.15	432.95	329.15	468.30	346.15	491.96
313.15	435.49	330.15	470.06	347.15	492.85
314.15	437.87	331.15	471.83	348.15	493.61
315.15	440.06	332.15	473.56	349.15	494.38
316.15	442.14	333.15	475.29	350.15	495.15
317.15	444.25	334.15	477.06	351.15	495.92
318.15	446.25	335.15	478.67	352.15	496.76
319.15	448.28	336.15	480.32	353.15	497.72
320.15	450.47	337.15	481.86	354.15	498.61
321.15	452.58	338.15	483.24	355.15	499.61
322.15	454.66	339.15	484.59		

^a^ Standard uncertainties of *u* (*T*) = 0.01 K and *u* (*p*) = 5 kPa, and the molar heat capacities *u* (*C*_p,m_) = 0.05 J·mol^−1^·K^−1^ both for MgB_4_O_7_·9H_2_O and MgB_6_O_10_·7.5H_2_O.

**Table 3:** Molar heat capacities obtained on the fitted Equations (1) and (2), as well as the thermodynamic functions (*H*_T_-*H*_298.15_), (*S*_T_-*S*_298.15_), and (*G*_T_-*G*_298.15_) for MgB_4_O_7_·9H_2_O and MgB_6_O_10_·7.5H_2_O from 306.15 to 355.15 K.

$\frac{T}{K}$	$\frac{Cp}{J\text{·}{\mathrm{mol}}^{-1}\text{·}K^{-1}}$	$\frac{H_{T}\;-\;H_{298.15}}{\mathrm{KJ}\text{·}{\mathrm{mol}}^{-1}}$	$\frac{S_{T}\;-\;S_{298.15}}{J\text{·}{\mathrm{mol}}^{-1}\text{·}K^{-1}}$	$\frac{G_{T}\;-\;G_{298.15}}{\mathrm{KJ}\text{·}{\mathrm{mol}}^{-1}}$
Hungchaoite, MgB_4_O_7_·9H_2_O				
298.15	400.86			
300.15	415.14	0.8161	2.7281	−0.0027
305.15	452.21	2.9743	9.8585	−0.0340
310.15	477.79	5.2814	17.3573	−0.1019
315.15	496.89	7.7153	25.1415	−0.2081
320.15	514.61	10.2557	33.1389	−0.3537
325.15	533.26	12.8850	41.2877	−0.5397
330.15	548.84	15.5875	49.5357	−0.7667
335.15	559.25	18.3498	57.8397	−1.0351
340.15	566.32	21.1609	66.1652	−1.3452
345.15	571.92	24.0119	74.4855	−1.6968
350.15	578.55	26.8960	82.7816	−2.0900
355.15	587.70	29.8090	91.0418	−2.5245
Mcallisterite, MgB_6_O_10_·7.5H_2_O				
298.15	387.81			
300.15	395.16	0.7830	2.6175	−0.0026
305.15	412.09	2.8020	9.2881	−0.0323
310.15	427.54	4.9007	16.1098	−0.0957
315.15	440.06	7.0701	23.0482	−0.1936
320.15	450.47	9.3016	30.0732	−0.3264
325.15	460.50	11.5876	37.1584	−0.4944
330.15	470.06	13.9213	44.2810	−0.6980
335.15	478.67	16.2966	51.4213	−0.9373
340.15	485.82	18.7081	58.5632	−1.2122
345.15	491.04	21.1511	65.6933	−1.5229
350.15	495.15	23.6221	72.8009	−1.8691
355.15	499.61	26.1179	79.8781	−2.2508
